# Missed manubriosternal dislocation in patient with thoracolumbar fracture, a case report

**DOI:** 10.1186/s12893-019-0564-y

**Published:** 2019-07-29

**Authors:** Wei-yu Jiang, Yun-lin Chen, Nan-jian Xu, Xu-dong Hu, Chao-yue Ruan, Wei-hu Ma

**Affiliations:** Department of Spine Surgery, Ningbo No.6 Hospital, No.1059, Zhongshan East Road, Ningbo, China

**Keywords:** Sternal fracture, Thoracolumbar chance fracture, Thoracic cage, Manubriosternal dislocation

## Abstract

**Background:**

Spine fractures combined with sternal injury are most commonly occur in the thoracic region. Lower cervical and thoracolumbar injuries have also been reported, especially for the patients with manubriosternal dislocation. The type of spine injury is easily recognized in initial presentation, but we may miss the sternal fracture and manubriosternal dislocation.

**Case presentation:**

A 23-year-old male patient complained with chest, right ankle, and lumbar pain after a fall at ground level, with diagnosis of right distal tibial fracture, sternal fracture, calcaneus fracture, and L2 vertebral fracture. However, neurologically he was completely normal. He underwent the operation for his lower extremity and spine, but we missed his manubriosternal dislocation after discharged. After one month, he came to the clinic with complained of chest pain, the imaging exams showed anterior dislocation of manubriosternal joint. We chose conservative treatment for manubriosternal dislocation. He was followed up at monthly intervals and radiographs along with computerized tomography showed satisfactory in fracture healing of lumber and the sternal fracture. However, the manubriosternal dislocation was malunioned. The patient had appearance deformity of the manubriosternal joint.

**Conclusion:**

This case supports the concept of the existence and clinical relevance of the thoracic cage theory, the thoracolumbar vertebrae should also be included in the thoracic cage theory.

## Background

Manubriosternal dislocations are rare, and may be related to spine fractures. High-energy traumas may result in severe injuries; and the most common area to be fractured is the thoracolumbar spine. However, when associated with sternal fractures, thoracic area is the most common. Lower cervical and thoracolumbar injuries are also reported, but very rare, especially for the patients combined with manubriosternal joint (MSJ) dislocation. The type of spine injury is easily recognized in initial examination, however, we may miss the sternal fracture and MSJ dislocation. Very few cases of thoracolumbar fracture combined with sternal fracture and MSJ dislocation have been reported in the literature. The objective of this case report is to present the occurrence of manubriosternal dislocation in a patient with thoracolumbar and sternal fracture, in which the clinical and radiological manifestation occurred only in the rehabilitation period.

## Case presentation

We present a 23-year-old male patient, who fell from a height of about 6 m. He felt chest, right ankle, and lumbar pain, and he was admitted to the local hospital where he was diagnosed with right distal tibial fracture, sternal fracture, calcaneus fracture, and L2 vertebral fracture. The primary management of patient was pain control and treating his edema. He was then transferred to our hospital.

**Fig. 1:**
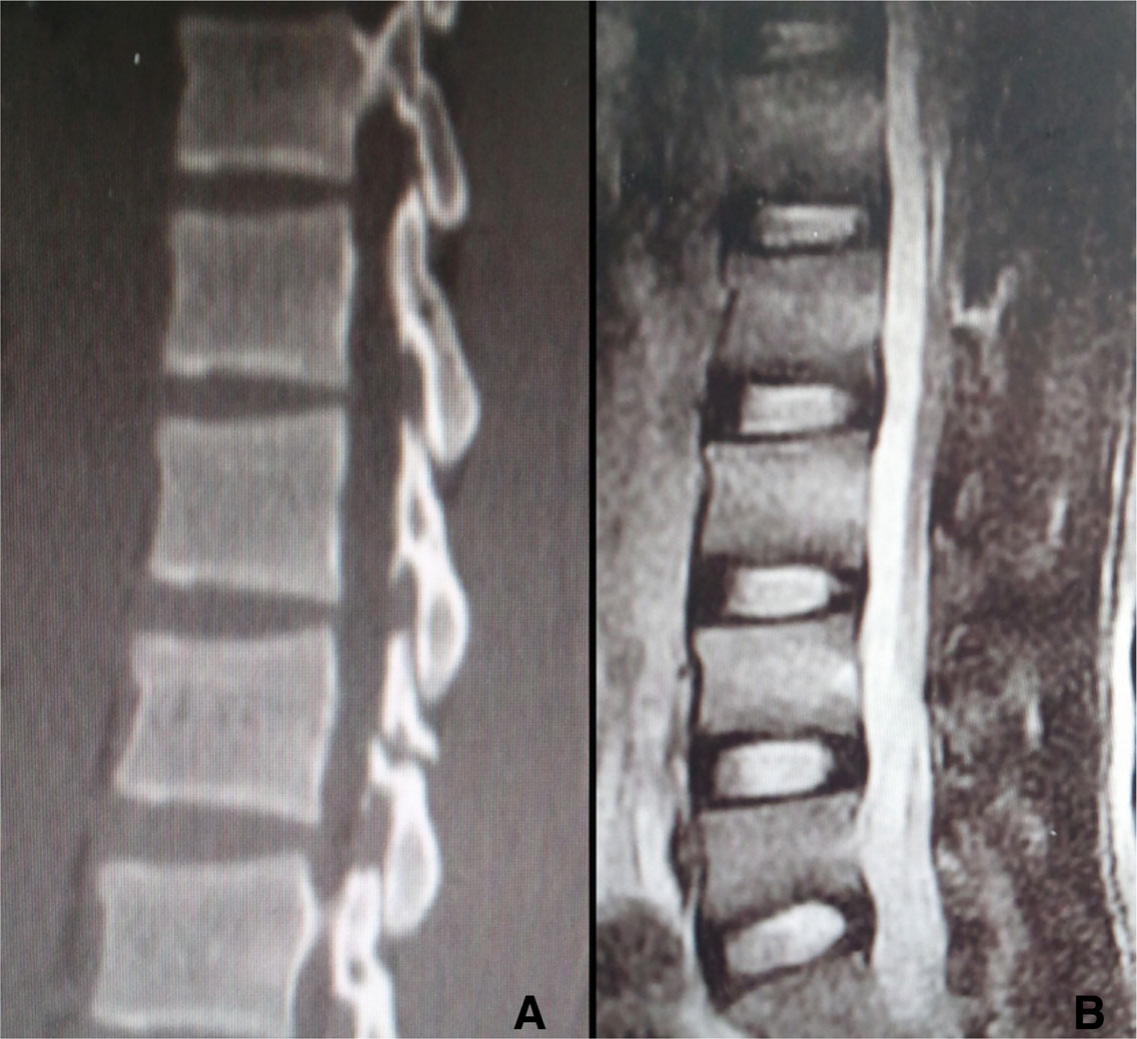
**a** Sagittal CT scan and **b** sagittal MRI showed L2 flextion-compression fracture

**Fig. 2:**
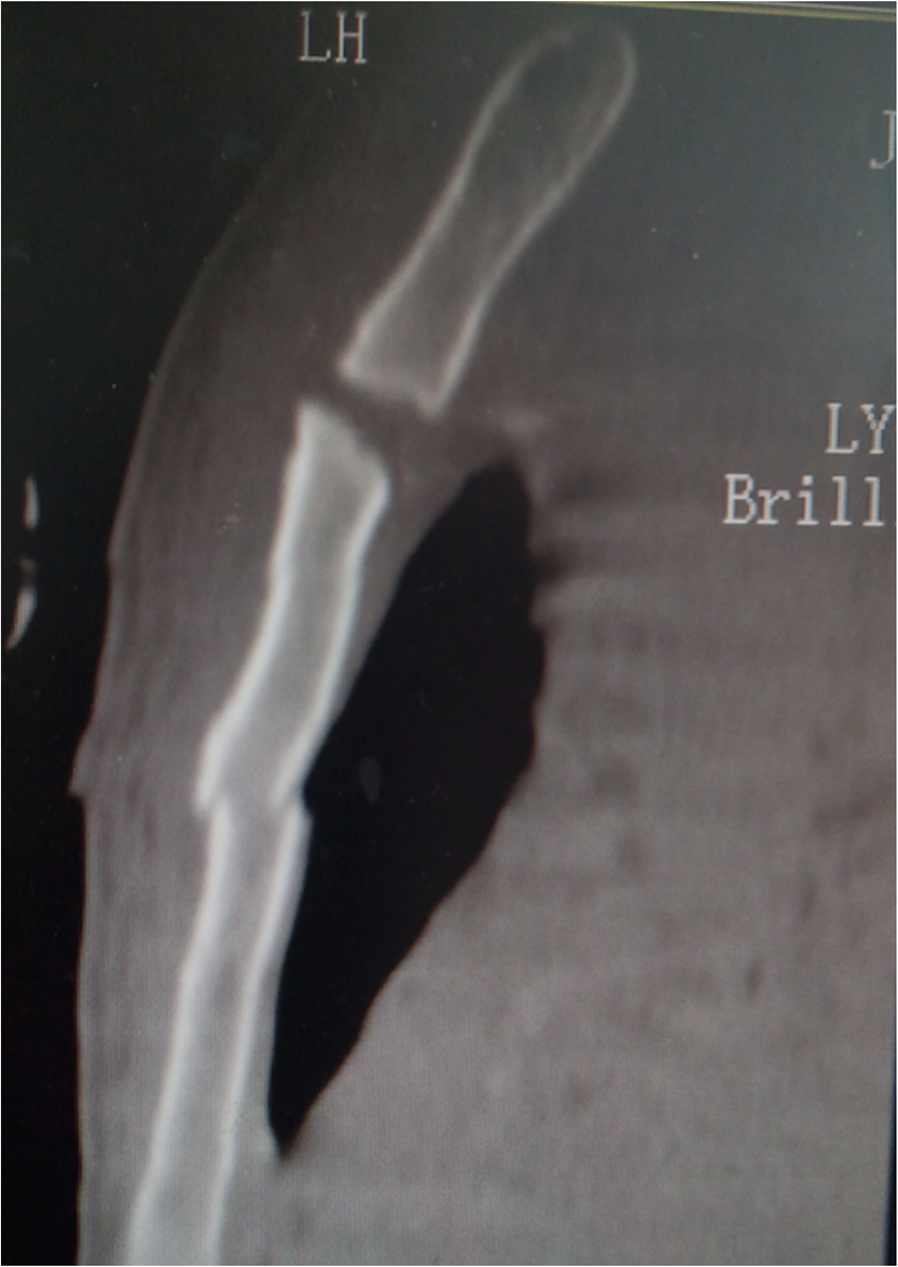
Sagittal CT scan of the sternum showing the sternal fracture without dislocation

**Fig. 3:**
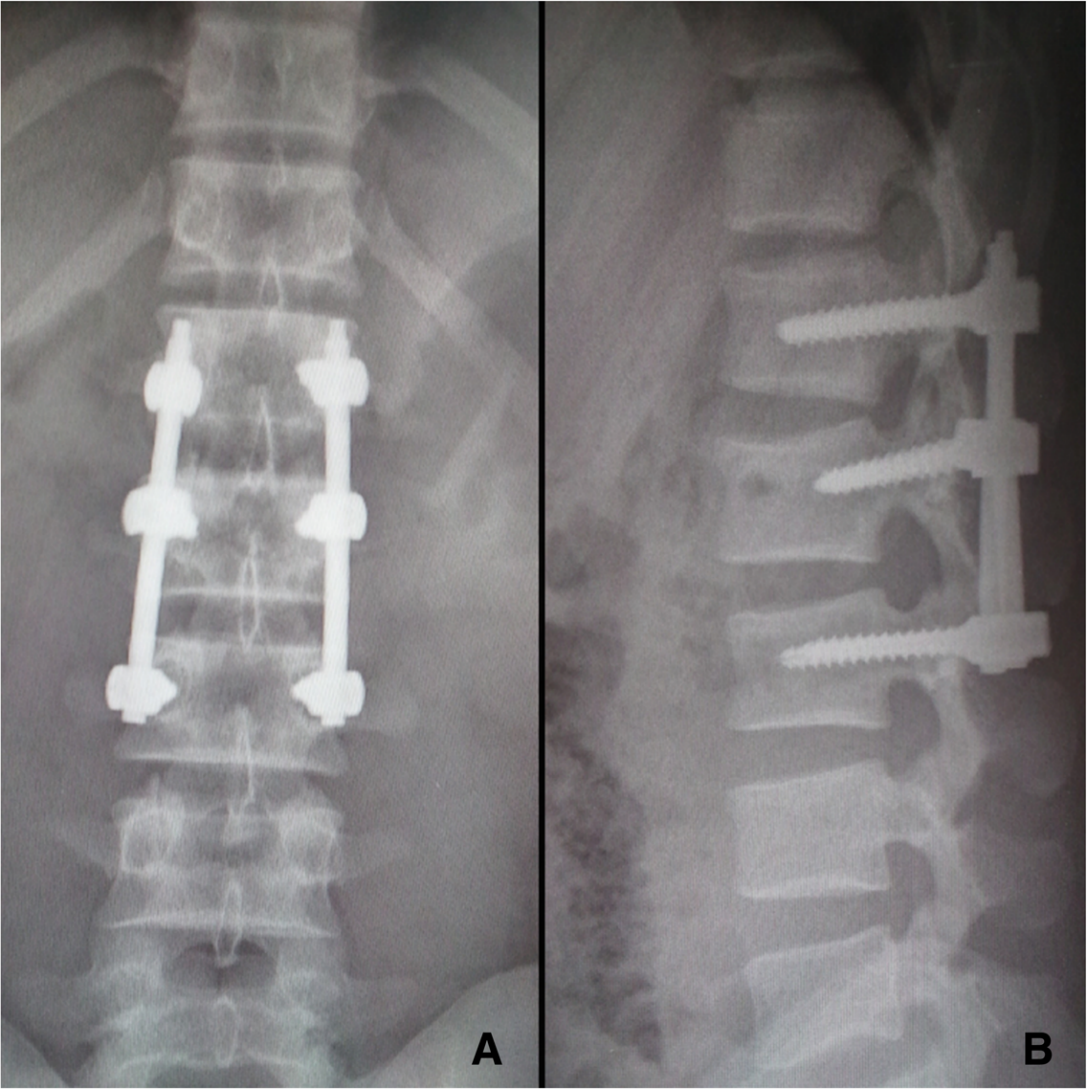
**a** Anteroposterior and **b** lateral radiograph of the lumbar spine showed the pedicel screws were inserted in the L1-3 vertebrae

**Fig. 4:**
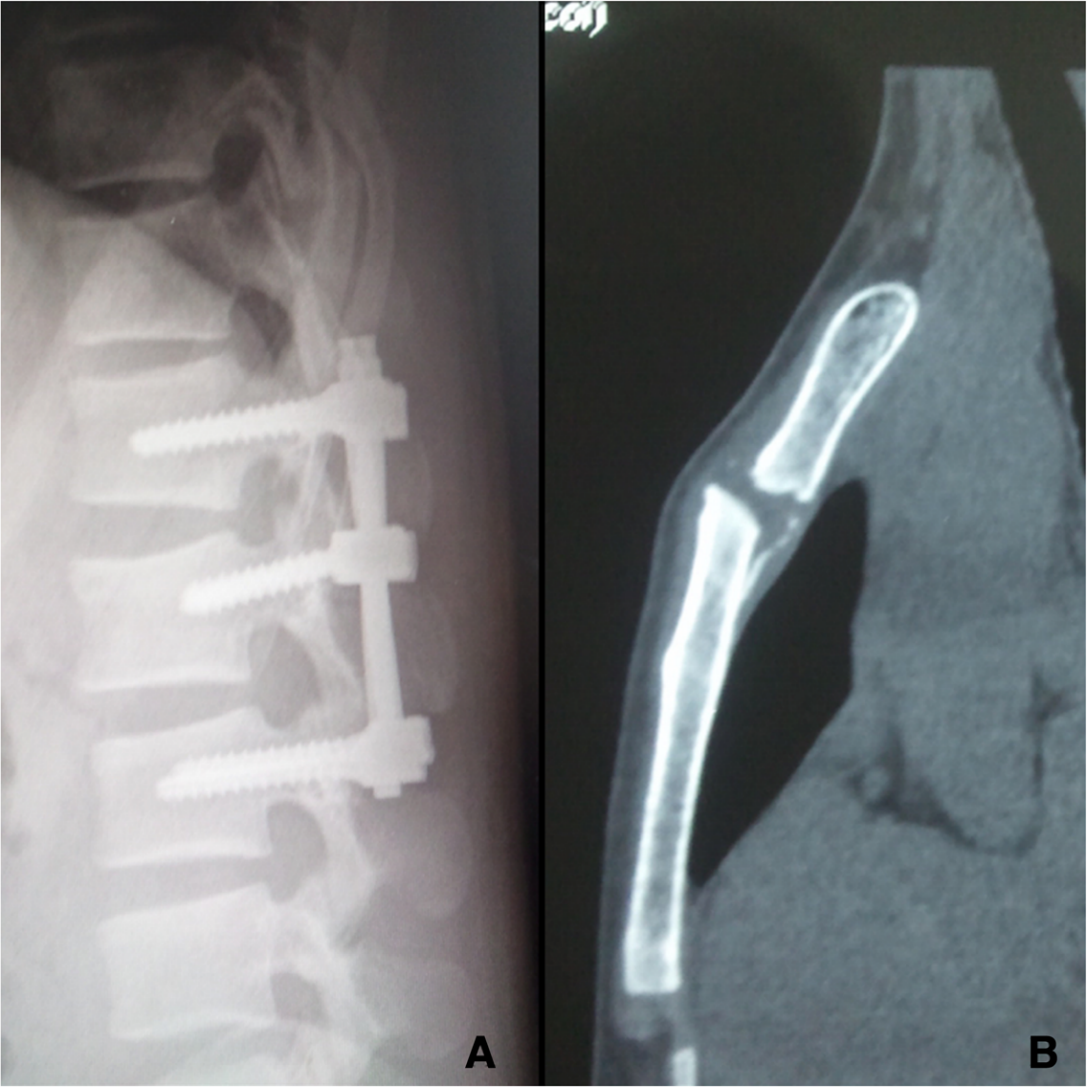
**a** Lateral radiograph showed fracture healing of the lumber. **b** sagittal CT scan of the sternum showed anterior dislocation of manubriosternal joint

**Fig. 5:**
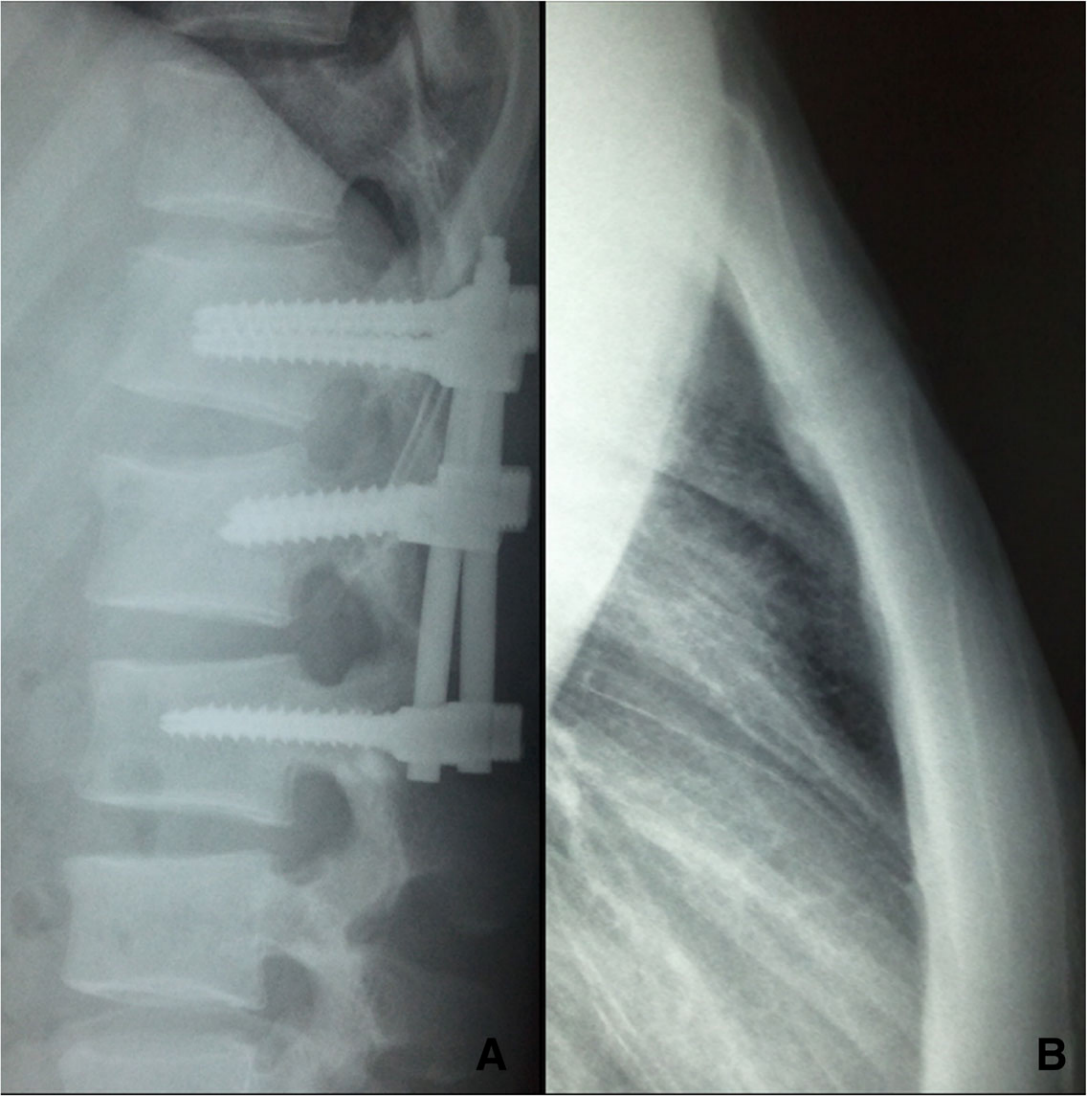
**a** Lateral radiograph showed fracture healing of the lumber and **b** malunion of manubriosternal dislocation, including appearance deformity of sternum

On examination, there was tenderness over the chest, right ankle, and lumber. There was no neurological deficit. Image studies showed right distal tibial fracture, calcaneus fracture, and L2 chance fracture accroding to the latest AO spine classification. Sagittal CT and MRI confirmed L2 chance fracture (Fig. 1). CT scan showed sternal fracture without dislocation (Fig. 2). Because this patient already had the whole lumbar spine MRI, we just performed a CT-scan of the thoracolumbar spine. The patient was scheduled for surgery after improvement in his general condition. The operation was performed with standard posterior midline incision; and the pedicle screws were inserted in the L1–3 (Fig. 3). The patient tolerated the operation well. There were no neurological complications. He was mobilized with the lumbar rigid orthosis on third postoperative day. After 1 month post-operatively, he came to the clinic complaining of chest pain, and X rays showed dislocation of the manubriosternal joint (Fig. 4). Since chest pain was not severe, and he could walk all by himself, we chose conservative treatment for manubriosternal dislocation. He was followed up at monthly intervals and radiographs along with computerized tomography showed satisfactory in fracture healing of lumber and the sternum. However, the manubriosternal dislocation was malunioned (Fig. 5). The patient appeared to have deformity of the manubriosternal joint in the final follow-up of 14 months. Since the patient had no limitations, he returned to his previous occupation.

## Discussion and conclusions

In 1993, Berg [1] present the concept of thoracic cage for the first time. Morgenstern and Watkins enhanced the thoracic cage theory that including the sternum, rib cage, and the thoracic spine, which has an inherent stability [2, 3]. Due to the existence of the thoracic cage, the stability of the thoracic vertebra is higher than other parts of the spine. Thoracolumbar fracture is more frequent due to relative immobility of thoracic spine compared to the lumbar spine.

Manubriosternal joint dislocation is very rare, representing 17.6% of lesions of the sternum and 0.5% of all traumatic injuries [4]. Manubriosternal joint dislocation is more likely to happen in patients with thoracic kyphosis and rheumatoid arthritis. Manubriosternal joint dislocations have been classified into two types according to the location of sternum, posterior dislocation (Type 1) or anterior dislocation (Type 2) [5]. In Type 1 dislocation, the body of the sternum dislocates dorsally; while in Type 2 dislocation, the body lies on the ventral side.

The association of the sternal and thoracic fractures has been reported, especially injuries caused by the flexion mechanism. When associated with sternal fractures, spine fractures most commonly occur in the thoracic region [6], however lower cervical and thoracolumbar injuries have also been reported in rare instances [6, 7]. Because sternal fracture displacement and manubriosternal joint dislocation mostly occur in the sagittal plane, lateral radiographs are more sensitive for identifying the injury. However, we may miss the diagonosis of manubriosternal dislocations if the patient took examination in supine position. The dislocation may also be reduct if the patient change their position.

In our case, the patient had L2 chance fracture, including the vertebral body and spinous process, indicated the hyperflexion mechanism. The powerful force is transmitted from the clavicles and ribs to the sternum, resulting in the fracture and dislocation of the sternum.

For manubriosternal joint dislocation, it was a type 2 with a flexion-distraction injury of the thoracolumbar spine. Type 2 dislocation mostly occurs from indirect trauma, as a result of hyperflexion or flexion-distraction injury of the spine. We made a summary of the literature that about patients who were diagosed with manubriosternal dislocation combined with spinal fractures (Table 1). In our table, most patients had type 2 dislocation of manubriosternal joint and thoracic fracture. Some studies recommended conservation treatment for sternal fracture and MSJ dislocation [7, 8, 13]; while others prefered to open reduction and fixation [5, 9–12]. In Jones’s study [9], they had patients combined with MSJ dislocation and lower cervical and lumbar fractures. Also they missed the spinal fractures for three patients. We recommend to pay more attention to sternum and the whole spine when patients had the hyper-flexion injury mechanism to rule out other fractures.

**Table 1:** Summary of related literatures

Author	Number	Cause	Type of dislocation	Location of spine	Treatment
Jenyo M.S. 1985 [8]	1	Road traffic accident	Type 2	T4	Cast
Jones et al. 1989 [9]	8	Flexion injury	Type 2	3 for thoracic level, 4 for lumbar level, 1 for lower cervical and thoracic level	1 patient had open reduction of MSJ, 3 patients missed the spinal fractures
Stahlman et al. 1995 [10]	1	Vehicle accident	Type 2	T5	Open reduction and fusion
Kalicke et al. 2006 [11]	1	Fall from the bicycle	Type 2	T6	MSJ fixation with plate
Labbe et al.2009 [7]	11	Car accident, fall and knocked over	Fracture-dislocation and subluxation	Upper thoracic	Conservation for sternal fractures
Herrero et al. 2011 [12]	1	Fall	Type 2	T9	Open reduction and fixation
Kothari et al. 2015 [13]	1	Fall	Type 2	T8–9	Conservation
Sarkeshik et al. 2019 [5]	1	Vehicle collision	Type 2	T6–7 fracture	MSJ fixation with plate

*MSJ* manubriosternal joint

The ribs played the primary role in transmitting the force from the spine to the sternum; with extrem flexion of the thoracic and thoracolumbar spine, the body of the sternum was forced upwards and forward by the lower ribs, therefore result in the sternum fracture. If the force is powerful enough, it may result in manubriosternal joint dislocation. Labbe’s study showed that the body of sternum was pushed in a proximal and ventral direction by the extreme flexion of the lumbar or thoracolumbar spine [7]. If the force was strong enough, it can lead to sternal fracture or manubriosternal joint dislocation. However, once the fracture occurs, the violence resulting into injury will tend to be more serious, which sometimes will involve multiple segments and often will be accompanied by the other injuries. In our case, the patient had right distal tibial fracture, sternal fracture, calcaneus fracture, and L2 chance fracture. Since dedicated sternum radiographs are not part of the standard trauma work-up, we may missed the sternal fracture and manubriosternal dislocation.

When we meet patients with spine fracture, especially the patients with thoracic or thoracolumbar fracture, we should pay more attention to the sternum. If the patient has a combined sternal fracture, we should try to prevent from manubriosternal dislocation. We think the thoracic cage theory should include the lower cervical and thoracolumbar spine.

Treatment for manubriosternal dislocation is argumentative. Conservative treatment includes observation and restricting sport activities, or closed reduction combined with immobilization. The conservative treatment is associated with higher rates of recurrent dislocation, and may result in chronic pain and progressive deformity [14]. However, some studies showed with good results. Patients treated with observation or manipulations to obtain the reduction of fracture and dislocation [15]. We chose observation and restricting sports activities for our patient. Surgical treatment is necessary if the conservative treatment fails.

The purpose of this case was to report on our experience with the diagnosis and treatment of patients with fracture of thoracolumbar and dislocation of manubriosternal joint. Despite its rarity, this lesion should be considered and close attention should be payed to the sternum, as was observed in our case.

Dislocation of manubriosternal joint is a very rare injury. High-energy traumas to the chest and spine may result in critical injuries, such as sternal fracture and dislocation, and thoracic or thoracolumbar fractures. Thoracic cage plays an important role in the stability of the thoracic spine. As the thoracic cage theory, we should pay attention to the rib cage, clavicle, sternum and spine if the patient had hyper-flexion injury mechanism. The lower cervical and thoracolumbar spine should also be included in the thoracic cage theory. In spinal fracture patients with suspected sternal injury, lateral radiographs including of the sternum should be done routinely. Detailed physical examination is also very important. If patient has tenderness of the sternum, even X ray or CT scan does not showed any fracture or dislocation, we should tell the patient he still has the risk of manubriosternal joint dislocation. Because the dislocation will be reduced when the patient was in supine position. If the patient’s sternal fracture was confirmed, conservative treatment can be performed with closed reduction combined with immobilization, or only by observation and restricting sport activities. Surgical treatment can be a good option if the reduction is not successful, or the instability continues after reduction of the manubriosternal joint.

## Data Availability

The datasets supporting the conclusion of this article are included within the article.

## Abbreviation

MSJ: Manubriosternal joint
